# Nurse managers experiences of their leadership roles in a specific mining primary healthcare service in the West Rand

**DOI:** 10.4102/curationis.v43i1.2129

**Published:** 2020-07-23

**Authors:** Sanele E. Nene, Hafisa Ally, Elizabeth Nkosi

**Affiliations:** 1 Department of Nursing, Faculty of Health Sciences, University of Johannesburg, Johannesburg, South Africa

**Keywords:** nurse managers, experiences, leadership roles, mining, primary healthcare

## Abstract

**Background:**

Nurse managers are leaders in mining primary healthcare. Their leadership roles include inspiring and empowering operational managers and nursing personnel, by leading with competence developing them to become followers with insight and direction. However, these leadership roles are not clearly defined, and are negatively influenced by the traditional mining leadership style.

**Objectives:**

The aim of this study was to explore and describe the nurse managers’ experiences of their leadership roles in a specific mining primary healthcare service on the West Rand, to develop recommendations to enhance these roles.

**Method:**

A qualitative, exploratory, descriptive and contextual research design was used in this study, following a phenomenological approach as a research method. A non-probability purposive sampling method was used. Nurse managers described experiences of their leadership roles during individual phenomenological interviews. Data saturation was reached on participant number 7. To analyse data, four stages of Giorgi’s descriptive phenomenological data analysis was used. An independent coder coded the data and a consensus meeting was held. The study was guided by the theoretical framework of Winkler’s role theory.

**Results:**

The following subthemes emanated from data analysis: (1) leadership role ambiguity, (2) leadership roles experienced and (3) challenges experienced in leadership roles.

**Conclusion:**

This study revealed that the leadership roles for nurse managers in a specific mining primary healthcare service are not clearly defined. Hence enhancements and expansions of these leadership roles remained stagnant. A clearly defined policy on leadership roles for nurse managers should be developed.

## Introduction

Leadership roles in mining Primary Healthcare (mPHC) service assist nurse managers (NMs) to resolve conflicts by engaging with their followers and other stakeholders, collectively leaning towards different solutions (Liphadzi, Aigbavboa & Thwala 2016:1; Okoroji, Anyanwu & Ukpere 2014:180). The purpose of Primary Health Care (PHC) at the mines in South Africa is to ensure that mine workers are healthy and fit to work underground for gold or mineral production (The South African Human Resources for Health Strategy 2012/2013–2016/2017:10).

To create a healthy and fit workforce the specific mining company in the study context has built the PHC services for the shafts for easy access to their employees. Caring for more than 17 000 mine employees, these PHC services include: medical and surgical services; emergency services at PHC level; health promotion; maternal care and women’s health; chronic disease; tuberculosis management; and human immunodeficiency virus or acquired immunodeficiency syndrome (HIV/AIDS) management (National Health Insurance 2015:32). Nurse Managers (NM) are leadership role players in this specific mPHC service. The unit managers are reporting to the NMs, and the NMs are reporting to the vice president of the mining company health and wellness division, which is overseeing all health-related roles and activities. Jooste (2014:29) defined leadership roles as the abilities to influence others in order to achieve shared organisational goals. It is an international mandate to have NMs as strong leadership role players in mPHC (Macphee et al. 2011:160; Zydziunaite & Souminen 2014:150).

The World Health Organization’s (2018:1) sustainable development goal number three aims to ensure a healthy living and well-being for all citizens. Hence NMs in PHC have a critical leadership role towards achieving this goal. Edward and Mbohwa (2013:125) reported that globalisation, economic downturn and other challenges facing NMs in mPHC in the 21st century, demand an evolutionary approach to leadership roles. This calls for NMs to explicitly understand their leadership roles, in order to lead with competence. The South African National Department of Heath Strategic Plan for Nursing Education, Training, and Practice (2011/2012–2016/2017:17) calls for strong leadership of role players in all nursing domains, including PHC. However, there is no clear description of leadership roles in this nursing strategy document.

In a study in PHC, Jooste and Hamani (2017:44) alluded to the fact that clarification and recognition of NMs’ leadership roles by the management should be a strategic exercise. McCallin and Frankson (2010:319) corroborate this view by stating that the roles of NMs have expanded in order for them to become leaders, and not just managers. Understanding of leadership roles of NMs in mPHC will help them to effectively perform their roles and use power and authority appropriately (Jooste 2014:35).

Hancock (2017) affirmed that NMs are key leadership agents expected to find common grounds to move mPHC forward and need to remain others-centred instead of being self-centred. Coetzee, Visagie and Ukpere (2014:831) emphasise that NMs’ leadership roles go beyond just implementing changes that result in better performance, but also involve guiding the followers in the direction of established organisational goals.

The understanding of NMs’ leadership roles in a mPHC will create an enabling and conducive environment, harvesting the achievement of shared organisational goals. Clarifying these leadership roles will assist NMs to practise directionally and with insight. As much as there is extensive literature on leadership roles, none of this literature is about mPHC. Exploration and description could lead to a better understanding and subsequent transformation and enhancement of leadership roles in mining, in order to improve employee engagement and commitment (Bezuidenhout & Schultz 2013:2). It is against this background that the researcher sought to explore and describe NMs’ experiences of their leadership roles in a specific mPHC service on the West Rand, in order to develop recommendations to enhance their leadership roles.

## Problem statement

Nurse Managers in a specific mPHC service demonstrated a lack of understanding of their leadership roles, despite the leadership development programme afforded to them by the employer. They were unable to exercise their leadership roles, which include effective communication of the organisational vision, inspiring and transferring enthusiasm to the followers (Jooste 2014:243; Pederson 2015:1). Traditional management styles and strong union influence are hampering the execution of leadership roles of NMs. According to the *Nursing Act* (33 of 2005) a NM can lead any healthcare establishment. Yet in this mPHC service, leadership roles of NMs are watered down, determined and informed by the mining management and unions, leaving them unsure of what exactly their role is. The mining management’s and unions’ voices in the PHC are louder than the voices of NMs, who should be leading in this context. This results in division between the NMs and their followers because they have to wait for instructions from the mining management and the unions before they communicate the decisions (Feather & Ebright 2013:64).

## Conceptual framework

**FIGURE 1:**
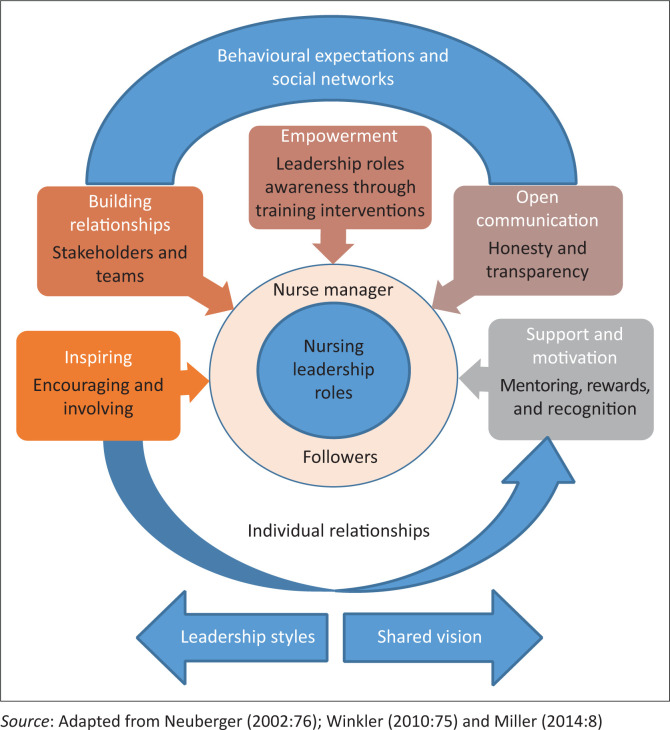
Conceptual framework of nurse managers’ leadership roles in a specific mining primary healthcare service.

This study was guided by Winkler’s leadership role theoretical framework (Neuberger 2002:76; Winkler 2010:75). In this theoretical framework, Neuberger (2002:76) and Winkler (2010:75) explained leadership roles using three components: (1) individual as being permanently influenced by behavioural expectations, (2) social networks and (3) relationships the individual is embedded in. Figure 1 illustrates a conceptual framework of leadership roles which was adopted from Miller (2014:8), depicting the elements embedded in Winkler’s (2010:75) theoretical framework:

Nurse Managers in mPHC are permanently influenced by behavioural expectations, social networks and therapeutic relationships created with individuals to effectively lead quality service delivery (Neuberger 2002:76; Winkler 2010:75). Behavioural expectations and social networks refer to the building of relationships, empowering followers, and ensuring transparent open communication in the organisation. Individual relationships refers to support, motivation and inspiration provided by the NMs to the followers using an appropriate leadership style such as a participative leadership to achieve the vision of the organisation (Miller 2014:8). The elements illustrated in the conceptual framework were confirmed in the findings of this study, as explained in subtheme 2: Leadership roles experienced.

## Aim and objectives of the study

The aim and objectives of this study were to explore and describe the NMs’ experiences of their leadership roles in a specific mPHC service at the West Rand, and to develop recommendations to enhance their leadership roles.

## Research design and method

### Research design

A qualitative, exploratory, descriptive and contextual research design was used to explore and describe the NMs’ experiences of their leadership roles in a specific mPHC service in the West Rand and to develop recommendations to enhance them. This design focuses on understanding through naturalistic observation rather that explaining and controlling measures, with subjective exploration of reality from the perspective of an insider as opposed to the outsider (De Vos 2011:308).

### Research method

This research was conducted in two phases, following the phenomenological approach. A phenomenological approach is a philosophical method used to explore and describe an individual’s experiences regarding a specific phenomenon (Ally 2017:19). Phase 1 involved the exploration and description of NMs’ experiences regarding their leadership roles in a specific mPHC service on the West Rand. In Phase 2, recommendations were derived from the findings of Phase 1, integrated with relevant literature.

#### Population and sampling

The population of this study consisted of 10 NMs, selected using a non-probability purposive sampling method. Purposive sampling refers to the researcher’s sampling of certain participants that will be included in the study, to best assist the researcher in understanding the research problem and question (Burns, Grove & Grey 2013:509; Creswell 2014:189). In this study data was saturated after interviewing seven participants. Data saturation is reached at the stage where no new themes emerge, only redundancy of data already collected (Burns & Grove 2011:317; Polit & Beck 2013:286).

## Data collection

Data was collected from the NMs using in-depth, phenomenological, individual interviews until data was saturated. The purpose of in-depth, phenomenological interviews was to explore and describe experiences of the NMs regarding the phenomenon under study. Finlay (2014:121) asserted that this data collection method assists the researcher to understand the participants’ experiences regarding the phenomenon. Data was collected by the researcher who is skilled in qualitative research, following interactive communication strategies. An appointment was made with the NMs to provide them with detailed information about the study so that they could decide whether or not they wished to participate. Voluntary informed consent forms for both interviews and audio recordings were signed by the participants.

The purpose of audio recording was indicated to the NMs, which was to save collected data in order to ensure verbatim transcriptions and to facilitate data analysis. The interviews were conducted according to the time schedule provided by each participant. Field notes were collected during each interview to make full and accurate descriptions of gestures and emotions expressed by the NMs. The following two open-ended research questions were posed to the participants: (1) What are your experiences of your leadership roles in this specific mPHC service on the West Rand? (2) What can be done to enhance your leadership roles in this specific mPHC service on the West Rand? Communication techniques such as clarification, probing, paraphrasing, reflecting, and bracketing were applied during the interviews. Interviews with the participants lasted approximately 30 to 45 minutes. The researcher stopped collecting data when no new information emerged from the NMs, affirming the saturation of data.

### Data analysis

Data analysis was conducted using Giorgi’s four stages of the phenomenological descriptive data analysis process, as cited by Holloway and Wheeler (2010:222–224). Recorded transcripts from the interviews were analysed and three subthemes emanated. An independent coder with vast expertise in phenomenological data analysis coded the data. A meeting to reach a consensus was held between the researcher and the independent coder.

### Trustworthiness

Lincoln and Guba’s criteria to establish trustworthiness in qualitative research was used in this study, constituting the following four constructs: credibility; transferability; dependability; and confirmability (in Polit & Beck 2013:584–597). The researcher ensured credibility through triangulation and prolonged engagement with the participants. Triangulation is the use of different data sources of information derived from examining evidence from the sources to build a coherent justification for themes (Creswell 2014:201). In-depth individual phenomenological interviews, a pilot study and capturing of field notes ensured that triangulation was achieved. A pilot study was conducted prior to data collection by interviewing one NM who met the inclusion criteria, but who did not form part of the sample. This was to ensure that the research questions are relevant for the selected population. Transferability was ensured by providing an adequate dense description of the setting in order to allow the prospective interested researcher to apply this study in other settings (Brink, Van der Walt & Van Rensburg 2014:173). By providing a dense description of the context of the study, research methods and participants, the researcher ensured that the study is dependable (Polit & Beck 2013:379). The researcher also reached consensus with the independent coder regarding the findings, and conducted a literature review of national and international publications to substantiate and confirm the results of the study.

### Ethical considerations

The following four ethical principles proposed by Dhai and McQuoid-Mason (2011:4–15) were applied in this study: autonomy, non-maleficence, beneficence, and justice. All participants provided informed consent in writing to participate in the study, and had the freedom to withdraw from the study, without any penalties being imposed on them, protecting them from discomfort and harm (Brink et al. 2014:35). The University of Johannesburg Ethics Committee, as well as the leadership of the organisation where the study was conducted provided permission to continue with the study (Reference: REC-01-73-2017). The benefits of the study were shared with the participants, which were the recommendations to enhance their experiences of leadership roles in their specific mPHC service. To achieve justice in this study, participants’ rights of freedom of choice, expression, and access to information were considered and protected.

## Discussion

Seven NMs from a specific mPHC participated in this study. They are all registered with the South African Nursing Council as professional nurses and nurse administrators, with more than one year’s experience in mPHC management. Their age ranged between 38 and 60 years, four were male, and three were female, six of them were black people and one was a white person.

The findings of the study revealed three subthemes as presented in Table 1. Some of the findings are presented below as verbatim quotes in italics and field notes are recorded in bold.

**TABLE 1:** Nurse managers’ experiences of their leadership roles in a specific mining primary healthcare service in the West Rand.

Number	Subthemes
1	Leadership role ambiguity
2	Leadership roles experienced
3	Challenges experienced in leadership roles

## Leadership role ambiguity

All participants demonstrated leadership role ambiguity by confusing leadership roles with managerial and clinical roles, as well as resource management. They mentioned management of equipment and material as being their leadership roles. The leadership role is to lead and coordinate the process of managing material and equipment resources to ensure that all resources required to deliver quality PHC are available.

The participants mentioned that:

‘We are all responsible for all the healthcare activities there.’ (Participant 2, Female, 50)

‘It’s a huge role at primary health to be honest with you, because it entails everything.’ (Participant 3, Female, 57)

In addition, another participant mentioned that:

‘That means all the activities that include health and health matters, we should be part of.’ (Participant 4, Male, 38)

Udlis and Mancuso (2015:276) propose that role ambiguity in leadership arises when expected leadership roles and values are not known by the management. The participants described their daily activities as their leadership roles. Lack of expectations, requirements, methods and information about leadership roles lead to confusion of leadership roles with clinical, managerial and resource management (Harijanto et al. 2013:100).

Viewing the managerial role as a leadership role, one participant lamented that:

‘[*The*] leadership role is more to do with management.’ (Participant 2, Female, 50)

Management and leadership roles are two distinct aspects with different characteristics, traits, and systems (Gumbo 2017:3; Mabelebele 2013:26). Jooste (2014:47) affirms that planning is the primary role of management, and not a leadership role. The experience of confusion between leadership and managerial roles varies widely, reflecting the rigidity or inflexibility of job-related expectations, including implementation of goals (Gaunter & Hansman 2017:118). Nurse Managers’ leadership roles entail using their energy to inspire people to be creative problem-solvers (Liphadzi, Aigbavboa & Thwala 2017:479).

Participants reported that:

‘Another leadership role for me is management of the followers, which is managing my staff.’ (Participant 2, Female, 50)

‘It covers all the aspects that you can think of in a setting, in a clinic – stock, everything, the tools that you use to collect data.’ (Participant 4, Male, 38)

Jooste (2014:285) alluded to the fact that NMs’ leadership roles include motivating and encouraging their followers in their daily activities. Skeepers and Mbohwa (2015:15) articulated that PHC NMs demonstrate their resource management roles by ensuring that stock, equipment and other resources are distributed and managed accordingly.

Another participant mentioned one of their leadership roles as a clinical role:

‘My main role is a clinical role, which is assessment, treatment and care.’ (Participant 5, Female, 40)

Bender (2016:33) reports that the clinical role in PHC only focuses on enabling the opportunity to transform the care environment at the point of practice and does not cover leadership roles. Nicol (2012:63) adds that clinical roles are concerned with the mind and routine, while leadership roles involve the capacity to align people to a common set of goals.

From the quotations and findings above, it can be concluded that a NM’s managerial role is to plan, organise, direct and control, while a leadership role is to inspire the followers, in order to achieve organisational goals as defined in the organisation’s strategic plan. These two should not be confused but can be integrated to achieve optimal results.

## Leadership roles experienced

The participants have experienced some leadership roles during their practice. These leadership roles were described by the participants as: being coordinators and facilitators of processes, engaging with stakeholders, supporting their teams, and empowering personnel.

Confirming the above statement, Participants commented that:

‘As the leader of the whole operation you have to make sure that […] you coordinate the activities of all the teams.’ (Participant 1, Male, 45)

‘The role of the manager is to coordinate all this so that we can give comprehensive healthcare to this individual.’ (Participant 2, Female, 50)

Another participant stated that:

‘Ja (yes), that is the main role of NSM, to coordinate and facilitate the activities of all the teams.’ (Participant 3, Female, 57)

Askerud and Conder (2017:424) articulated that leadership in mPHC is expected to collaborate and coordinate the PHC processes, including facilitation of information to promote the quality and efficacy of required patient care. Coordination and facilitation of processes among team members in a mPHC service is difficult to observe without active involvement of NMs in all teams’ activities (Chang et al. 2017:926; Jooste 2014:348). Coordination and facilitation of processes requires NMs who are self-driven, enthusiastic, and self-starters. Coordination of activities is essential for NMs in a mPHC setting, because it allows NMs to facilitate multiple interdependent tasks routinely performed by different team members (Bogdanovic et al. 2015:2).

Participants said that one of their leadership roles is stakeholder engagement and team support, as confirmed by the following statement:

‘We call it stakeholder engagement. We engage with almost weekly or daily with different stakeholders.’ (Participant 4, Male, 38)

Another participant affirmed that:

‘Another stakeholder is [*the*] National Department of Mineral Resources as our main regulator, that is my engagement role in this position.’ (Participant 2, Female, 50)

One participant mentioned that:

‘The team that is working on the ground is supported to understand the systems that we are using in the company, you support and advise accordingly.’ (Participant 6, Female, 43)

Esguerra, Beck and Lidskog (2017:62) report that engagement of different and relevant stakeholders in the mPHC is a critical leadership role in a mining PHC service. There have been explicit calls for relevant stakeholder engagements from a global level to a national level to make scientific knowledge relevant and usable in the leadership field (Esguerra et al. 2017:59).

Dyess et al. (2016:10) claim that NMs expect to be supported by the mining health management so that they are able to also support their followers, executing their leadership roles effectively. Chang et al. (2017:916) attest that it is the NMs’ leadership role to provide support to staff during the process of coordination and facilitation. From the quotations and literature provided above, it is evident that coordinating and facilitating processes in this mPHC service is one of the experiences of NMs.

All participants indicated that they are expected to empower their personnel to ensure that activities are well executed in this mPHC. This is confirmed by the following quotations from the participants:

‘It start[*s*] from training, that means we need to empower people from the lower ranks as they grow up.’ (Participant 1, Male, 45)

‘You include even development, because you still need to develop people, empowerment of personnel (using both hands to explain).‘ (Participant 4, Male, 38)

‘Training is very key, and one need[*s*] to be very strong and understand the vision of the company.’ (Participant 7 Male, 59)

In their PHC study, Jooste and Ntamane (2014:227) attest that NMs have the leadership role of creating an empowered nursing profession that supports the successful practice of existing and future generations of followers in a mPHC service. By providing nursing personnel with development opportunities to strengthen their independence, NMs in mPHC are effectively exercising their empowerment and autonomous leadership roles (Peiter, De Melo Lanzoni & De Oliveira 2016:821). Jooste (2014:359) affirmed that PHC NMs are mandated to create an empowering environment in which followers are motivated and encouraged to learn and perform to their full potential.

## Challenges experienced in leadership roles

Participants reported that they experienced challenges regarding their leadership roles. These challenges were discussed as: lack of effective communication, bureaucratic mining management and union influence.

Participants mentioned that they experienced communication problems, and this created a gap affecting the PHC service delivery. The following quotations confirm this finding:

‘I think a bit of gap that we have at this stage, I think it’s communication, there is always a problem with communication (participant used both hands to explain).‘ (Participant 2, Female, 50)

‘There is always a problem with communication (participant used both hands to explain).’ (Participant 4, Male, 38)

‘And then the person that you report to didn’t disclose everything, so you [*are*] always lagging with communication at primary healthcare.’ (Participant 6, Female, 43)

‘So you [*are*] always lagging with communication at primary healthcare.’ (Participant 7, Male, 59)

There is a lack of effective communication in the mPHC services that NMs are expected to resolve through dynamic and continuous interactions among themselves and other stakeholders (Woodward, More & Van der Heyden 2016:20). Napier et al. (2018:120) reported that failure to recognise the communication gap in a mining PHC setting is a serious leadership role challenge, which results in personnel frustration. Price-Down (2018:171) affirmed that PHC NMs cannot execute their leadership roles efficiently without excellent communication.

Napier et al. (2018:134) and Dobbs (2016:30) propose that transparent leaders disclose critical information to their followers as early as possible to build and promote trust, eliminating communication challenges. Woodward et al. (2016:42) confirmed that transparent leadership that discloses information encourages employees to be expressive and to become active listeners, which minimises communication challenges. Effective communication is a critical domain that needs to be well facilitated from strategic to operational management in the mPHC service to ensure that clients are receiving the quality care they deserve.

Participants mentioned that they experienced bureaucratic directives from the mining management, and this was impeding the execution of their leadership roles execution.

Confirming this are the following quotations from the participants:

*‘*We are expected to implement without taking into consideration, the constraints, the frustrations, the practicalities of all of that.’ (Participant 4, Male, 38)

‘They just sit on the strategic level making strategic decisions.’ (Participant 3, Female, 57)

Crossler et al. (2017:50); and Obolonsky (2017:587) asserted that the biggest leadership challenge was that NMs were expected to function within bureaucratic organisational rules and this leads to frustrations. Hemel (2014:1) claims that bureaucracy forces NMs to operate according to decisions that are taken at strategic level, making it impossible for them to deal with operational challenges affecting their leadership roles.

Participants reported that:

‘The policy must inform our health model, not the model to inform the policy. There are some changes in policies that you need to amend.’ (Participant 5, Female, 40)

‘And I must make sure that they comply to whatever company policy.’ (Participant 7, Male, 59)

Lawlis, Knox and Jamieson (2016:43) state that NMs pushed their followers to comply with the company’s policies, even if they were aware that the policy did not address the status quo. Zhang et al. (2014:1217) stated that the operating health model in a mPHC service should provide all role players with opportunities to make decisions that assess the impact of policies. Peltzer et al. (2015:121) claimed that providing NMs in a mining PHC service with opportunities to influence policies ensured that policies were relevant and aligned to the health model.

Unions are playing a critical role in the mPHC service, representing members, protecting their rights, and promoting their members’ interests. Nurse Managers commented that the influence of unions is too strong, and it is challenging their leadership roles. This is confirmed by the following quotations from the participants:

‘Ja (yes), it becomes a challenge, I think because we also working with a highly unionised environment.’ (Participant 1, Male, 45)

‘Another challenging stakeholder [*is*] organised labour; they are our internal stakeholders (crossing legs).’ (Participant 3, Female, 57)

Wohlgemuth (2017:57) states that management considers unions as a challenge in a highly unionised environment such as mPHC. Lane and Perozzi (2014:35) agreed that unions are perceived as a problem by organisations’ leaders.

Organised labour only focuses on the employees’ interests, which might be challenging for the NMs in mPHC, because their leadership roles are driven by skills, quality and team competency (Spehar et al. 2017:110).

One participant also added that:

‘A challenge is when the union is saying, “I don’t agree with one, two, three, four, five”.’ (Participant 1, Male, 45)

Labour unions in mPHC challenge NMs during the execution of leadership roles, questioning the working conditions of peripheral mine workers (Dorigatti 2017:938).

## Limitations

As this was a qualitative and contextual study, only the NMs’ experiences regarding their leadership roles in a specific mPHC service on the West Rand were described, therefore the findings cannot be generalised to the entire mining PHC industry.

## Recommendations

From the results of this study which were provided as three subthemes, the recommendations for nursing practice and policy, nursing education and research were made. This was Phase 2 of the study.

### Nursing practice and policy

The development of a clearly defined policy on NMs’ leadership roles in a mPHC service is recommended. Creation of an environment that is conducive to open and honest communication regarding NMs’ leadership roles in a mPHC service should be considered. The researcher also recommends developing a good working relationship with the unions to collectively deal with leadership role challenges experienced by NMs. Fortunato, Gigliotti and Ruben (2017:200) concluded that NMs should focus on the development and maintenance of strong relationships with unions, so that they are able to collectively predict, recognise, detect, and address challenges that may rise to crisis levels.

### Nursing research

The study on the leadership role experiences should be extended to the NMs in public PHC clinics, public hospitals and private hospitals. Research on the same phenomenon should be performed in other mPHC services around the country. A quantitative study on a similar topic should be done to provide a broader perspective on NMs’ experiences regarding their leadership roles in a mPHC service.

### Nursing education

This study can be used as an empowerment tool for NMs in their leadership roles, and to facilitate the continuing professional development on leadership roles of NMs. Jooste and Ntamane (2014:227) pointed out that NMs have the leadership role of creating an empowered nursing profession that supports the successful practice of existing and future generations of followers in a mPHC service. The findings of this study can be integrated with the nursing management curriculum and NMs’ development programmes.

## Conclusion

This study revealed that the leadership roles for NMs in the mPHC are not clearly defined. This resulted in NMs not knowing what exactly was expected from them as leadership role players. Hence enhancements and expansions of these leadership roles remain stagnant. The study findings will enable the participants to address their experiences of leadership role ambiguity and leadership challenges. The recommendations will ensure that experienced leadership roles are enhanced and expanded.
